# Effectivness of specific mobile health applications (mHealth-apps) in gestational diabtetes mellitus: a systematic review

**DOI:** 10.1186/s12884-021-04274-7

**Published:** 2021-12-05

**Authors:** Claudia Eberle, Maxine Loehnert, Stefanie Stichling

**Affiliations:** Medicine with specialization in Internal Medicine and General Medicine, Hochschule Fulda - University of Applied Sciences, Leipziger Strasse 123, 36037 Fulda, Germany

**Keywords:** Gestational diabetes mellitus, Medical apps, mHealth-apps, mHealth, Pregnancy, Smartphones

## Abstract

**Background:**

Gestational diabetes mellitus (GDM) emerges worldwide and is closely associated with short- and long-term health issues in women and their offspring, such as pregnancy and birth complications respectively comorbidities, Type 2 Diabetes (T2D), metabolic syndrome as well as cardiovascular diseases. Against this background, mobile health applications (mHealth-Apps) do open up new possibilities to improve the management of GDM. Therefore, we analyzed the clinical effectiveness of specific mHealth-Apps on clinical health-related short and long-term outcomes in mother and child.

**Methods:**

A systematic literature search in Medline (PubMed), Cochrane Library, Embase, CINAHL and Web of Science Core Collection databases as well as Google Scholar was performed. We selected studies published 2008 to 2020 analyzing women diagnosed with GDM using specific mHealth-Apps. Controlled clinical trials (CCT) and randomized controlled trials (RCT) were included. Study quality was assessed using the Effective Public Health Practice Project (EPHPP) tool.

**Results:**

In total, *n* = 6 publications (*n* = 5 RCTs, *n* = 1 CCT; and *n* = 4 moderate, *n* = 2 weak quality), analyzing *n* = 408 GDM patients in the intervention and *n* = 405 in the control groups, were included. Compared to control groups, fasting blood glucose, 2-h postprandial blood glucose, off target blood glucose measurements, delivery mode (more vaginal deliveries and fewer (emergency) caesarean sections) and patient compliance showed improving trends.

**Conclusion:**

mHealth-Apps might improve health-related outcomes, particularly glycemic control, in the management of GDM. Further studies need to be done in more detail.

**Supplementary Information:**

The online version contains supplementary material available at 10.1186/s12884-021-04274-7.

## Background

Gestational diabetes mellitus (GDM) emerges worldwide. In 2019 and with reference to the International Diabetes Federation (IDF), approx. 20 million or approx. 16% of live births had some form of hyperglycemia in pregnancy, approx. 84% were diagnosed with GDM [1]. Globally, the prevalence of GDM ranged between approx. 2.1% and approx. 37.5% in 2019 [2]. Risk factors leading to GDM include maternal age, obesity, gestational weight gain [3, 4]. GDM is associated with adverse pregnancy and birth outcomes for mother and child, eg, increased risks of preeclampsia, caesarean sections, macrosomia and shoulder dystocia [3, 5].

Having the concept of transgenerational programming (“fetal or perinatal programming”) in mind, intra-uterine exposure to hyperglycemia “programs” the offspring to eg, obesity, glucose intolerance, T2D, insulin resistance, metabolic syndrome, high blood pressure, and cardiovascular diseases [6–10]. In this context, early strategies are needed to improve the management of GDM effectively. Mobile health (mHealth), especially mobile health applications (mHealth-Apps), open up innovative strategies to improve clinical outcomes in the management of GDM [11]. mHealth defines the support of medical procedures and health care measures through movile devices such as smartphones [12]. mHealth-Apps allow a more specific and individual management of patient care [13]. We recently analyzed the usage behavior of GDM related mHealth-Apps und their components [11, 14, 15]. Although smartphone apps may be beneficial in self-management, there is still a lack of individualized diabetes therapy during pregnancy [11, 13–15]. There is evidence that smartphone-supported GDM management may improve glycemic control and compliance of GDM patients and may decrease the numbers of “off-target” measurements as well as insulin dosages and complication rates in gestational diabetics and their children [11, 16]. For example, Xie et al. [17] examined telemedical interventions in GDM patients, including mHealth apps and other (mostly web-based) applications, and found an improvement in clinical outcomes such as glycemic control. We systematically reviewed studies that evaluated the effectiveness of GDM specific mHealth-Apps on health-related outcomes in GDM patients compared to control groups.

## Methods

### Search strategy and eligibility criteria

We performed a systematic search in Medline (PubMed), Cochrane Library, Embase, CINAHL and Web of Science Core Collection databases based on the “Preferred Reporting Items for Systematic Reviews and Meta-Analyses (PRISMA)” guidelines [18]. The search strategy included the following keywords: “smartphone” OR “mobile phone” OR “cell phone” OR “iOS” OR “android” AND “mobile application” OR “app” AND “gestational diabetes mellitus”. For example, the strategy in PubMed was as follows: ((“Diabetes, Gestational”[Mesh]) AND (((((“Smartphone”[Mesh]) OR (“Cell Phone”[Mesh])) OR (“mobile phone”[Title/Abstract])) OR (ios [Title/Abstract])) OR (android [Title/Abstract]))) AND ((app [Title/Abstract]) OR (“Mobile Applications”[Mesh])). We manually searched reference lists and Google Scholar to identify further studies.

We included studies that evaluated mHealth-Apps in GDM management compared to usual care (control group), and reported clinical parameters such as glycemic control, pregnancy, birth and neonatal outcomes. We involved peer-reviewed randomized controlled trials and clinical controlled trials. We have filtered the studies by year (January 2008 to November 2020) and by language (German and English). The studies were screened and selected by two independent reviewers. Moreover, we excluded poster, comments, study protocols, duplicates and studies addressing GDM diagnosis and prevention.

After removing duplicates, we scanned the titles and abstracts of the remaining studies, and reviewed the full texts.

### Data extraction and analysis

We extracted author, year, design, intervention and control group, sample size, main findings related to the outcomes of interest, and significances. The results were structured by outcomes: glycemic control outcomes (1), pregnancy and birth related outcomes (2), and neonatal outcomes (3).

### Risk of bias assessment

Risk of bias was assessed using the Effective Public Health Practice Project (EPHPP) tool [19, 20]. The tool consists of the following components: selection bias, study design, confounders, blinding, data collection methods, and withdrawals [19]. EPHPP is a validated instrument to assess studies with health-related topics and appropriate for quantitative intervention studies. The tool rates the study quality as strong, moderate or weak.

## Results

### Study characteristics

Overall, *n* = 121 records were identified through database and manual searching. After removing duplicates, we screening *n* = 72 titles and abstracts and excluded *n* = 60 unsuitable papers. The reasons for exclusion are documented in the PRISMA flowchart (Multimedia Appendix 1). After assessing *n* = 12 papers with full-text, *n* = 6 inappropriate studies were excluded. Finally, we included n = 6 eligible studies in this systematic review analyzing *n* = 408 GDM patients in the intervention and *n* = 405 in the control groups. These studies were divided into *n* = 5 two-arm randomized controlled trials (RCT) [16, 21–24] and *n* = 1 controlled clinical trial (CCT) [25]. Additionally, in the CCT, a group of *n* = 50 women without GDM was included as a second control group [25]. Multimedia Appendix 2 gives an overview of included studies regarding study design, number of participants, main features of the investigated mHealt-apps, and main outcomes. We structured the outcomes in glycemic control, pregnancy- and birth-related as well as neonatal outcomes.

Four of the studies reported the majority of their participants being in the third trimester of pregnancy (mean gestational age in intervention groups: 29.1 (±1.9) weeks [22], 30.9 (±3.6) weeks [24], 31.2 (±4.1) weeks [23] and *n* = 88 (76.4%) being between 25th and 32nd gestational week [21]). Two studies reported only the values of gestational age used as inclusion criterion (gestational age: < 34 weeks [16], between 14 + 0 and 34 + 6 [25]). Mean maternal age differed in the studies between 31.2 (±4.1) [23] and 33.9 (±5.5) years [24].

The risk of bias assessment using EPHPP tool is allocated in Multimedia Appendix 3. The quality of four studies was rated as “moderate” [16, 21, 23, 24], .whereas the quality of the two remaining studies was rated as “weak” [22, 25]. The relevant component that was crucial for low quality ratings was the lack of blinding.

### Glycemic control outcomes

Multimedia Appendix 4 displays the summary of the results regarding glycemic control outcomes.

Glycated Hemoglobin A_1c_ (HbA_1c_) (*n* = 164 intervention group (IG), *n* = 161 control group (CG)). Overall, the intervention patients showed better HbA_1c_ values than the control groups [23, 24]. Guo et al. displayed a significant difference in favor of the intervention group (− 1.3% intervention group versus − 0.6% control group, *P < .*001) [23], .whilst Mackillop et al. recognized a slight increase in both groups (0.02% per 28 days in the intervention group verses 0.03% per 28 days in the control group, 95% CI − 0.05 to 0.03) [24].

Fasting blood glucose (FBG) (*n* = 69 IG, *n* = 62 CG). Significant improvements of FBG were found by Yang et al. (*P < .*001) and Bromuri et al. (*P < .*001) favoring the intervention group [22, 25].

1-h Postprandial blood glucose (PBG) (*n* = 57 IG, *n* = 50 CG). Yang et al. displayed lower, but not significantly, PBG values in the intervention compared to control group (7.71 ± 0.73 mmol/L IG verses 7.75 ± 2.08 mmol/L CG, *P = .*780) [25].

2-h Postprandial blood glucose (PBG) (*n* = 69 IG, *n* = 62 CG). There were significant improvements of 2 h-PBG values after the interventions [22, 25]. Yang et al. showed a significant difference in favor of the intervention group (*P < .*001) [25]. Bromuri et al. reported significant differences for morning (*P < .*001) and noon (*P < .*001) favoring the intervention group [22].

2-h Oral Glucose Tolerance Test (OGTT) (*n* = 176 IG, *n* = 181 CG). Neither Guo (*P = .*638) et al. nor Borgen et al. (*P = .*22) found significant differences between groups [21, 23].

Mean blood glucose (*n* = 170 IG, *n* = 157 CG). There were significant improvements regarding mean blood glucose [Bromuri et al. (*P < .*001) and Miremberg et al. (*P < .*001)] [16, 22]. However, Mackillop et al. found improved blood glucose values, but no significant differences between groups regarding the rate of change of blood glucose (− 0.16 mmol/L in intervention group versus − 0.14 mmol/L in control group, *P = .*78) [24].

Patient compliance (ratio between actual and instructed blood glucose measurements × 100) (*n* = 124 IG, *n* = 120 CG). Significant improvements were shown in both studies [Guo et al. (*P < .*001) and Miremberg et al. (*P < .*001)] [16, 23].

Off-target FBG measurements (n = 124 IG, n = 120 CG). Miremberg et al. (*P < .*001) as well as Guo et al. (*P < .*001) displayed significant differences in favor of the intervention group [16, 23].

Off-target 1 h-PBG measurements (*n* = 60 IG, n = 60 CG). Miremberg et al. found significant differences in favor of the intervention group (*P < .*001) [16].

Off-target 2 h-PBG measurements (*n* = 64 IG, n = 60 CG). displaying significant differences favoring the intervention group (*P < .*001) [23].

### Pregnancy- and birth-related outcomes

The findings of pregnancy and birth related outcomes are displayed in Multimedia Appendix 5.

Pregnancy-induced hypertension and/or preeclampsia (*n* = 218 IG, *n* = 212 CG). Although the intervention groups showed lower rates regarding the outcome, no significant differences were found by Mackillop et al. (*P = .*22), Miremberg et al. (*P > .*99) or Yang et al. (*P = .*347).

Preterm birth (*n* = 158 IG, *n* = 152 CG). There were fewer preterm births in the intervention groups, but neither Yang et al. (*P = .*248) nor Mackillop et al. (*P > .*05) did find significant differences.

Induction of labor (*n* = 172 IG, *n* = 181 CG). Though one study reported fewer incidents of induction of labor in the intervention group [21], no significant differences were found by Borgen et al. (*P = .*33) or Miremberg et al. (*P = .*248) [16, 21].

Shoulder dystocia (*n* = 220 IG, *n* = 222 CG). In all three studies only one incident of shoulder dystocia occurred [16, 23, 24].

Mode of delivery (*n* = 390 IG, *n* = 393 CG). Borgen et al. displayed significant differences (*P = .*03) and found obviously fewer operative vaginal deliveries and emergency caesarean sections, and more spontaneous vaginal deliveries and planned caesarean sections in the intervention group [21]. Mackillop et al. reported significant differences (*P = .*005) as well and recognized more vaginal deliveries and a lower rate of caesarean sections, especially of emergency caesarean sections, in the intervention group [24]. Guo et al., Miremberg et al. and Yang et al. did not find any significant differences [16, 23, 25].

### Neonatal outcomes

The results regarding these outcomes are shown in Multimedia Appendix 6.

Birth weight. The results showed a trend towards lower birth weight in the intervention groups, but no significant differences were found by Borgen et al. (*P = .*69), Mackillop et al. (*P = .*18), Miremberg et al. (*P = .*878) or Yang et al. (*P = .*988) [16, 21, 24, 25].

Macrosomia. Fewer incidents of macrosomia occurred in the intervention groups compared with the control groups, but no significant differences were found by Guo et al. (*P = .*295) or Yang et al. (*P = .*542) [23, 25].

Large for gestational age (LGA). Mackillop et al. did not report the concrete number of incidents and did not find significant differences (*P > .*05), whereas Miremberg et al. reported the same amount of LGA in the intervention and control group (*P > .*99) [16, 24].

Hypoglycemia (*n* = 277 IG, *n* = 263 CG). Fewer incidents of hypoglycemia of the newborn were reported favoring the intervention groups but not significant [23, 25]. Yang et al. did not report on significance in this outcome [25]. No significant differences were found by Guo et al. (*P = .*185), Mackillop et al. (*P = .*42) or Miremberg et al. (*P > .*99) [16, 23, 24].

Admission to higher level of care (*n* = 330 IG, n = 330 CG). The studies showed a trend towards being fewer often transferred to neonatal intensive care units favoring the intervention group. Neither Borgen et al. (*P = .*38), Mackillop et al. (*P = .*08), Miremberg et al. (*P > .*99) nor Yang et al. (*P = .*657) found significant differences between groups [16, 21, 24, 25].

## Discussion

In total, especially glycemic control and pregnancy and birth related outcomes showed improving trends through using GDM specific mHealth-apps. In detail, compared to control groups, fasting blood glucose, 2-h postprandial blood glucose, off target blood glucose measurements, delivery mode (more vaginal deliveries and fewer (emergency) caesarean sections) and patient compliance improved by using mHealth-apps in GDM care. We found only a few RCTs and CCTs evaluating the clinical effectiveness of GDM specific mHealth-Apps. Despite the limited data, the included studies indicated that GDM specific mHealth-Apps show a trend towards improving the management of GDM.

Especially more data in terms of parameters that stick out the most in terms of diabetic but also pregnancy/maternity as well as neonatal health (eg, HbA_1c_, FBG, PBG, OGTT, oral glucose challenge test (OGCT) [26]) are needed. However, in our review each of those parameters was only investigated in one or two studies, except for the OGCT. A standardized set of investigated outcomes is needed to extend the evidence on the effectiveness of GDM specific mHealth-Apps. In addition to that, nutritional and physical activity outcomes should be anlayzed as well, since these are basic treatments in terms of the management and control of GDM [27–29].

We assume that mHealth-apps enhance the compliance, empower the patients, and enable a more intensive and closely monitored therapy.

### Glycemic control parameters

Overall, the results showed an obvious trend in improving glycemic control parameters. Both studies, that evaluated HbA_1c_, displayed lower HbA_1c_ values respectively a lower rate of change in the intervention group [23, 24]. FBG results show an improvement through GDM specific mHealth-Apps, both studies reporting significant differences between the groups [22, 25]. But the quality of both studies was rated as weak. Regarding the PBG, findings on 1-h after glucose or nutrition administration are lacking. None of the studies reported on the OGTT values 60 min after glucose administration and only one study included the 1 h-PBG [25]. But Bromuri et al. and Yang et al. found significant 2 h-PBG improvements in their intervention groups [22, 25]. Though, the results are restricted by the fact that quality of both studies was rated as weak. Moreover, an effect on OGTT value 120 min after glucose administration needs further investigation. Improvements were displayed in the intervention and control groups and none of the studies found significant differences between groups [21, 23]. Furthermore, the results displayed a clear effect of GDM specific mHealth-Apps on mean blood glucose values, since all studies found improvements in their intervention groups [16, 22, 24]. In addition, an improving trend of patient compliance could be observed, since Guo et al. and Miremberg et al. found significant improvements and got a study quality rating as moderate [16, 23]. Moreover, there is a positive trend in reducing off-target measurements in terms of FBG, 1 h-PBG and 2 h-PBG [16, 23].

### Pregnancy- and birth-related outcomes

Mode of delivery was the only pregnancy and birth-related outcome in which significant differences were found [21, 24]. However, against this background the use of a GDM specific mHealth-App may improve GDM management and leads to fewer emergency caesarean sections and more vaginal deliveries [21, 24].

Moreover, a positive effect on induction of labor, preterm birth, pregnancy-induced hypertension and/or preeclampsia might be possible. Although none of the studies reported significant differences regarding one of these outcomes, most of them showed improved rates of incidents in the intervention compared with control groups [16, 21, 23–25]. No trend regarding shoulder dystocia can be derived, since the outcome occurred only once in one study, even though three studies included shoulder dystocia as an outcome [16, 23, 24].

### Neonatal outcomes

The use of GDM specific mHealth-Apps may improve birth weight or incidence of macrosomia. The studies found no significant differences [16, 21, 23–25]. But some studies showed a promising trend towards lower birth weight or fewer incidents of macrosomia in the intervention groups [16, 21, 23, 25].

In addition to that and more importantly, the results display a trend towards infants being fewer often transferred to neonatal intensive care units by using mHealth-apps in GDM care [16, 21, 24, 25]. No clear trend regarding hypoglycemia of the newborn can be derived [23–25].

### Comparison with prior work

Skar et al. found that a GDM specific mHealth-App can increase the confidence of women with GDM in their self-management and improve their motivation for behavioral changes [30]. Moreover, Chen et al. conclude in their scoping review that GDM specific mHealth-Apps can provide time- and cost-efficient personalized interventions to improve GDM management [31]. Although it is not shown in the included studies, an improved GDM management can reduce the risks of LGA, shoulder dystocia or hypertensive disorders [32, 33]. In addition, Xie et al. [17] noted that telemedicine interventions (including app-based interventions) can decrease the glycemic levels of GDM patients effectively and also reduce the risk of maternal, fetal and neonatal complications.

Other studies that did not specifically examine apps, but rather telemedical web and internet-based interventions as part of GDM management, showed promising trends.

For example, Rasekaba et al. [34] concluded that glycemic control indicated an improving trend in favor of telemedicine. The authors indicated advantages of telemetric systems in the reduction of face-to-face and unscheduled consultations. Moreover, Ming et al. [35] analyzed telemedicine technologies for diabetes in pregnancy showing a significant HbA_1c_ reduction of − 1.14% (mean difference) (95% CI: 0.25 to 0.04).

### Limitations

There are some limitations that should be noted: the available literature is very limited and further limited by discrepancies between investigated outcomes. The included studies were published relatively recently, the oldest being from 2016. This suggest that the topic has become more and more important over the past years. Because of that, we expect more publications soon. Heterogeneity in the investigated outcomes show that a more standardized set of outcomes, especially regarding glycemic control parameters is needed. In addition to that, the quality of the included papers is moderate to weak. There are only a few studies in the area of GDM and apps; due to the small number, the results should be viewed with caution.

## Conclusions

Since pregnant women tend to use and accept mHealth-Apps as part of accompanying pregnancy, further research on the full potential of GDM specific mHealth-Apps is needed [14, 15]. mHealth-Apps improved the management of other types of diabetes mellitus as well [36, 37].

In total, especially glycemic control outcomes showed improving trends through using GDM specific mHealth-apps. Fasting blood glucose, 2-h postprandial blood glucose, off target blood glucose measurements, delivery mode (more vaginal deliveries and fewer (emergency) caesarean sections) and patient compliance enhanced by using mHealth-apps in GDM management. Despite the limited data, mHealth-Apps showed potential to improve the management of GDM.

## Supplementary Information

**Additional file 1: Figure 1**: PRISMA flowchart. **Table 1**: Summary of included studies. **Table 2**: Appraisal of included studies. **Table 3**: Glycemic control outcomes. **Table 4**: Pregnancy and birth outcomes. **Table 5**: Neonatal outcomes.

## Data Availability

All data generated and analyzed during are included within the article and its supplementary information files.

## Abbreviations

CCT: Controlled clinical trial
CG: Control group
EPHPP: Effective Public Health Practice Project
FBG: Fasting blood glucose
GDM: Gestational diabetes mellitus
HbA_1c_: Glycated hemoglobin A_1c_
IG: Intervention group
MA: Meta-analysis
mHealth: Mobile Health
MQ: Moderate quality
PRISMA: Preferred Reporting Items for Systematic Reviews and Meta-Analyses
RCT: Randomized controlled trial
T2D: Type 2 diabetes mellitus
WQ: Weak quality
