# Supplementary material for: Effectivness of specific mobile health applications (mHealth-apps) in gestational diabtetes mellitus: a systematic review
Source: BMC Pregnancy Childbirth. 2021 Dec 5;21:808. doi: 10.1186/s12884-021-04274-7 (PMC8645100; doi:10.1186/s12884-021-04274-7)
Supplement: Supplementary file 1 — Additional file 1: Figure 1: PRISMA flowchart. Table 1: Summary of included studies. Table 2: Appraisal of included studies. Table 3: Glycemic control outcomes. Table 4: Pregnancy and birth outcomes. Table 5: Neonatal outcomes. [file 12884_2021_4274_MOESM1_ESM.docx]

**SUPPLEMENTARY INFORMATION**

EFFECTIVNESS OF SPECIFIC MOBILE HEALTH APPLICATIONS
(mHEALTH-APPS) IN GESTATIONAL DIABTETES MELLITUS:
A SYSTEMATIC REVIEW

Claudia Eberle, M.D.^1^, Maxine Loehnert, M.Sc. ^1^, Stefanie Stichling, M.Sc.^1^

**Table of Contents**

Figure 1: PRISMA flowchart 2

Table 1: Summary of included studies. 3-4

Table 2: Appraisal of included studies. 5

Table 3: Glycemic control outcomes. 6-7

Table 4: Pregnancy and birth outcomes. 8-10

Table 5: Neonatal outcomes. 11-12

**Figure 1: PRISMA flowchart (adapted from Moher et al. 2009).**

Studies included in final synthesis (n = 6)

## Identification

## Eligibility

Full-text articles assessed for eligibility
(n = 12)

## Included

Records screened
(n = 72)

## Screening

Records after duplicates removed
(n = 72)

Records excluded
(n = 60)

Wrong topic/ intervention
(n = 28)

Wrong design (poster, study protocols, proceedings papers, no control group etc.) (n = 23)

Not GDM patients (n = 9)

Full-text articles excluded
(n = 6)

Other outcomes (not health-related) (n=6)

Additional records identified through reference lists/ Google Schoolar (n = 1)

Records identified through database searching
(n = 121)

**Table 1: Summary of included studies.**

| **Reference** | **Study design** | **Participants** | **Included Outcomes** | **Main Features of the app** |
| --- | --- | --- | --- | --- |
| Borgen et al. 2019^26^ | 2 arm RCT | intervention group: n=112  control group: n=121 | - **Glycemic Control Outcomes:** OGTT-120min - **Pregnancy- and Birth related Outcomes:** Induction of Labor, Mode of Delivery - **Neonatal Outcomes:** Apgar Score After 5 min, Birth Weight, Transfer to Higher Level of Care (Newborn), Infant Nutrition in the First week, Cessation of Breast Feeding | Education, Diary/Tracing, Feedback on Blood Glucose Values |
| Guo et al. 2019^28^ | 2 arm RCT | intervention group: n=64  control group: n=60 | - **Glycemic Control Outcomes:** HbA1c at Diagnosis, HbA1c at Delivery, OGTT-fasting, OGTT-120min, Off-Target FBG Measurement, Off-Target 2h-PBG Measurements, Patient Compliance - **Pregnancy- and Birth related Outcomes:** Shoulder Dystocia, Mode of Delivery, Frequency of Outpatient Service, Episiotomy - **Neonatal Outcomes:** Hypoglycemia of the Newborn, Macrosomia | Diary/Tracing, Link/Data Transfer to Web Interface for HCPs, Contact to HCP, Feedback on Blood Glucose, Education |
| Mackillop et al. 2018^29^ | 2 arm RCT | intervention group: n=103  control group: n=102 | - **Glycemic Control Outcomes:** HbA1c, Blood Glucose (rate of change), Blood Glucose Readings/Day - **Pregnancy- and Birth related Outcomes:** Preterm Birth, Mode of Delivery, Maternal Weight Gain, Maternal Pregnancy-Induced Hypertension or Preeclampsia, Transfer to Higher Level of Care (Mother), Shoulder Dystocia, Transition to Hypoglycemic Medication During Study, Major Perineal Trauma, Delivery Less Than 37 Weeks - **Neonatal Outcomes:** Transfer to Higher Level of Care (Newborn), Birth Weight, LGA, Neonatal Hypoglycemia, Neonatal Jaundice | Diary/Tracing, Link/Data Transfer to Web Interface for HCPs, Feedback on Blood Glucose, Reminder, Education |
| Miremberg et al. 2018^22^ | 2 arm RCT | intervention group: n=60  control group: n=60 | - **Glycemic Control Outcomes:** Patient Compliance, Mean Blood Glucose, Off-Target FBG Measurements, Off-Target 1h-PBG Measurements - **Pregnancy- and Birth related Outcomes:** Preeclampsia, Gestational Hypertension, Induction of Labor, Mode of Delivery, Shoulder Dystocia, Polyhydramnios, Third- or Fourth-Degree Perineal Tears - **Neonatal Outcomes:** Birth Weight, LGA, Transfer to Higher Level of Care (Newborn), Hypoglycemia of the Newborn, Respiratory Morbidity, Phototherapy, Neonatal Death, Composite Adverse Neonatal Outcome | Diary/Tracing, Link/Data Transfer to HCPs, Feedback on Blood Glucose, Education, Contact to HCP |
| Bromuri et al. 2016^27^ | 2 arm RCT | intervention group: n=12  control group: n=12 | - **Glycemic Control Outcomes:** Mean Blood Glucose, FBG, Preprandial Blood Glucose, 2h-PBG | Diary/Tracing, Link/Data Transfer to Web Interface for HCPs |
| Yang et al. 2018^30^ | CCT | intervention group: n=57  control group (GDM, no intervention): n=50  control group (no GDM): n=50 | - **Glycemic Control Outcomes:** FBG, 1h-PBG, 2h-PBG - **Pregnancy- and Birth related Outcomes:** Pregnancy-Induced Hypertension, Mode of Delivery, Premature Rupture of Fetal Membranes, Premature Delivery - **Neonatal Outcomes:** Birth Weight, Head Circumference, Fetal Length, Macrosomia, Neonatal Hypoglycemia, Neonatal Hyperbilirubinemia, Transfer to Higher Level of Care (Newborn) | Diary/Tracing, Education, Link/Data Transfer to Web Interface for HCPs |

CCT=controlled clinical trial, FBG=fasting blood glucose, GDM=gestational diabetes mellitus, HbA1c=Glycated Hemoglobin A1c, HCP=health care professionals, LGA=large for gestational age, OGTT=oral glucose tolerance test, PBG=postprandial blood glucose, RCT=randomized clinical trial

**Table 2: Appraisal of included studies using Effective Public Health Practice Project (EPHPP) tool.**

|  | **Component Ratings** | | | | | | **Global Rating** |
| --- | --- | --- | --- | --- | --- | --- | --- |
| **Reference** | **Selection Bias** | **Study Design** | **Confounders** | **Blinding** | **Data Collection Methods** | **Withdrawals and Drop-Outs** |  |
| Borgen et al. 2019 | Strong | Strong | Weak | Moderate | Moderate | Strong | Moderate |
| Bromuri et al. 2016 | Moderate | Strong | Strong | Weak | Weak | Strong | Weak |
| Guo et al. 2019 | Moderate | Strong | Strong | Moderate | Weak | Strong | Moderate |
| Mackillop et al. 2018 | Strong | Strong | Strong | Weak | Strong | Strong | Moderate |
| Miremberg et al. 2018 | Strong | Strong | Strong | Moderate | Weak | Strong | Moderate |
| Yang et al. 2018 | Moderate | Strong | Strong | Moderate | Weak | Weak | Weak |

**Table 3: Glycemic control outcomes.**

| **Outcome** | **Characteristics of Outcome, unit** | **Reference** | **Kind of group** | **Baseline** | **Follow up** | **Significance** |
| --- | --- | --- | --- | --- | --- | --- |
| **HbA1c** | [%] | Guo et al. 2019^28^ | intervention (n=64) | 6.0 (0.4) | 4.7 (0.2) | **p<0.001** |
|  |  |  | control (n=60) | 5.9 (0.3) | 5.3 (0.3) |  |
|  |  | Mackillop et al. 2018^29^ | intervention (n=100) | 5.42 (0.34) | NR* | p>0.05 |
|  |  |  | control (n=101) | 5.39 (0.35) | NR* |  |
| **FBG** | [mmol/L] | Yang et al. 2018^30^ | intervention (n=57) | NR | 4.31 (0.75) | **p<0.001** |
|  |  |  | control (n=50) | NR | 5.31 (1.29) |  |
|  | Morning, [mmol/L] | Bromuri et al. 2016^27^ | intervention (n=12) | NR | 4.6 (4.3-4.9) | **p<0.001** |
|  |  |  | control (n=12) | NR | 4.8 (4.5-5.2) |  |
|  | Preprandial Noon, [mmol/L] |  | intervention (n=12) | NR | 5 (4.2-6.1) | p=0.08 |
|  |  |  | control (n=12) | NR | 5.4 (4.7-6.4) |  |
|  | Preprandial Evening, [mmol/L] |  | intervention (n=12) | NR | 4.7 (4.3-5.5) | **p<0.001** |
|  |  |  | control (n=12) | NR | NR |  |
| **OGTT-120** **min** | [mmol/L] | Guo et al.2019^28^ | intervention (n=64) | 9.7 (1.3) | 7.0 (1.6) | p=0.683 |
|  |  |  | control (n=60) | 9.9 (1.5) | 7.1 (1.5) |  |
|  |  | Borgen et al. 2019^26^ | intervention (n=112) | NR | 6.7 (6.2 to 7.1) | p=0.22 |
|  |  |  | control (n=121) | NR | 6.0 (5.6 to 6.3) |  |
| **1h-PBG** | [mmol/L] | Yang et al. 2018^30^ | intervention (n=57) | NR | 7.71 (0.73) | p=0.780 |
|  |  |  | control (n=50) | NR | 7.75 (2.08) |  |
| **2h-PBG** | [mmol/L] | Yang et al. 2018^30^ | intervention (n=57) | NR | 5.76 (0.67) | **p<0.001** |
|  |  |  | control (n=50) | NR | 6.94 (2.47) |  |
|  | Morning, [mmol/L] | Bromuri et al. 2016^27^ | intervention (n=12) | NR | 5.7 (4.9-6.5) | **p<0.001** |
|  |  |  | control (n=12) | NR | 6.1 (5.3-6.8) |  |
|  | Noon, [mmol/L] |  | intervention (n=12) | NR | 5.9 (5.2-6.6) | **p<0.001** |
|  |  |  | control (n=12) | NR | 6.3 (5.6-7.3) |  |
|  | Evening, [mmol/L] |  | intervention (n=12) | NR | 6.3 (5.6-7) | p=0.59 |
|  |  |  | control (n=12) | NR | NR |  |
| **Mean Blood Glucose** | [mmol/L] | Bromuri et al. 2016^27^ | intervention (n=12) | NR | 5.4 (4.7-6.4) | **p<0.001** |
|  |  |  | control (n=12) | NR | 5.7 (4.9-6.7) |  |
|  | [mg/dL] | Miremberg et al. 2018^22^ | intervention (n=60) | NR | 105.1 (8.6) | **p<0.001** |
|  |  |  | control (n=60) | NR | 112.6 (7.4) |  |
|  | Rate of change, [mmol/L] | Mackillop et al. 2018^29^ | intervention (n=98) | NR | NR | p=0.78 |
|  |  |  | control (n=85) | NR | NR |  |
| **Off-Target Blood Glucose Measurements** | FBG [%] | Miremberg et al. 2018^22^ | intervention (n=60) | NR | 4.7 (0.4) | **p<0.001** |
|  |  |  | control (n=60) | NR | 8.4 (0.6) |  |
|  |  | Guo et al. 2019^28^ | intervention (n=64) | NR | 4.6 (0.4) | **p<0.001** |
|  |  |  | control (n=60) | NR | 8.3 (0.6) |  |
|  | 1h-PBG [%] | Miremberg et al. 2018^22^ | intervention (n=60) | NR | 7.7 (0.8) | **p<0.001** |
|  |  |  | control (n=60) | NR | 14.3 (0.8) |  |
|  | 2h-PBG [%] | Guo et al. 2019^28^ | intervention (n=64) | NR | 7.9 (0.7) | **p<0.001** |
|  |  |  | control (n=60) | NR | 14.7 (0.8) |  |
| **Patient Compliance** | [%] | Miremberg et al. 2018^22^ | intervention (n=60) | NR | 84 (0.16) | **p<0.001** |
|  |  |  | control (n=60) | NR | 66 (0.28) |  |
|  |  | Guo et al. 2019^28^ | intervention (n=64) | NR | 83.3 (12.5) | **p<0.001** |
|  |  |  | control (n=60) | NR | 70.4 (10.1) |  |

All data shown as number (%) or mean (standard deviation or 95% confidence interval); results considered significant p<0.05

FBG=fasting blood glucose, GDM=gestational diabetes mellitus, HbA1c=Glycated Hemoglobin A1c, NR=not reported, OGTT=oral glucose tolerance test, PBG=postprandial blood glucose

*No specific follow up values were reported, but mean 0.02% rise per 28 days in intervention group and mean 0.03% rise per 28 days in the control group was noticed

**Table 4: Pregnancy and birth outcomes.**

| **Outcome/Reference** | **Characteristics of Outcome, unit** | **Intervention group** | **n(total) intervention group** | **Control group(s)** | **n(total) control group** | **Significance** |
| --- | --- | --- | --- | --- | --- | --- |
| **Induction of Labor** | | | | | | |
| Borgen et al. 2019 ^26^ | Yes, n (%) | 54 (48.2) | n=112 | 66 (54.5) | n=121 | p=0.33 |
|  | No, n (%) | 58 (51.8) |  | 55 (45.5) |  |  |
| Miremberg et al. 2018 ^22^ | n (%) | 24 (40) | n=60 | 17 (28.8) | n=60 | p=0.248 |
| **Preterm Birth** | | | | | | |
| Yang et al. 2018 ^30^ | n (%) | 1 (1.75) | n=57 | 3 (6.0), no GDM  6 (12.0), GDM | n=50, no GDM  n=50, GDM | p=0.248 |
| Mackillop et al. 2018 ^29^ | n (%) | 5 (5.0) | n=101 | 13 (12.7) | n=102 | p>0.05 |
| **Shoulder Dystocia** | | | | | | |
| Guo et al. 2019 ^28^ | n (%) | 0 | n=64 | 0 | n=60 | p>0.99 |
| Mackillop et al. 2018 ^29^ | n (%) | 1 (1.0) | n=100 | 0 (0) | n=102 | p=0.99 |
| Miremberg et al. 2018 ^22^ | n (%) | 0 | n=60 | 0 | n=60 | p>0.99 |
| **Pregnancy induced hypertension and/or preeclampsia** | | | | | | |
| Yang et al. 2018 ^30^ | n (%) | 1 (1.75) | n=57 | 0 (0.0), no GDM  4 (8.0), GDM | n=50  n=50 | p=0.347 |
| Mackillop et al. 2018 ^29^ | n (%) | 1 (1.0) | n=101 | 5 (4.9) | n=102 | p=0.22 |
| Miremberg et al. 2018 ^22^ | n (%) | 0 | n=60 | 1 (1.7) | n=60 | p>0.99 |
| **Mode of delivery** | | | | | | |
| Yang et al. 2018 ^30^ | Cesarean Section, n (%) | 18 (31.58) | n=57 | 11 (22.0), no GDM  7 (14.0), GDM | n=50, no GDM  n=50, GDM | p=0.266 |
|  | Vaginal Delivery, n (%) | 37 (64.91) |  | 39 (78.0), no GDM  40 (80.0), GDM |  | p=0.136 |
|  | Vacuum Extraction, n (%) | 2 (3.51) |  | 0 (0.0), no GDM  3 (6.0), GDM |  | p=0.181 |
| Mackillop et al. 2018 ^29^ | vaginal, n (%) | 52 (51.4) | n=101 | 42 (41.2) | n=102 | **p=0.005** |
|  | caesarean elective, n (%) | 21 (20.8) |  | 23 (22.5) |  |  |
|  | caesarean emergency, n (%) | 6 (5.9) |  | 24 (23.5) |  |  |
|  | assisted, n (%) | 22 (21.8) |  | 13 (12.7) |  |  |
| Miremberg et al. 2018 ^22^ | Normal vaginal delivery, n (%) | 48 (80) | n=60 | 40 (67.7) | n=60 | p=0.147 |
|  | Instrumental delivery, n (%) | 4 (6.7) |  | 1 (1.7) |  | p=0.364 |
|  | Cesarean delivery, n (%) | 12 (20) |  | 20 (33.3) |  | p=0.147 |
|  | Emergent cesarean delivery, n (%) | 4 (6.7) |  | 7 (11.6) |  | p=0.528 |
| Borgen et al. 2019 ^26^ | Spontaneous vaginal delivery, n (%) | 81 (71.7) | n=112 | 75 (61.5) | n=121 | **p=0.03** |
|  | Operative vaginal delivery, n (%) | 9 (8.0) |  | 11 (9.0) |  |  |
|  | Planned caesarean section, n (%) | 12 (10.6) |  | 8 (6.6) |  |  |
|  | Emergency caesarean section, n (%) | 10 (8.8) |  | 27 (22.1) |  |  |
| Guo et al. 2019 ^28^ | normal vaginal delivery, n (%) | 48 (75) | n=64 | 40 (66.7) | n=60 | p=0.142 |
|  | instrumental delivery, n (%) | 3 (4.6) |  | 4 (6.7) |  | p=0.364 |
|  | cesarean delivery, n (%) | 20 (33.3) |  | 16 (25.0) |  | p=0.352 |

All data shown as number (%) or mean (standard deviation or 95% confidence interval); results considered significant p<0.05

GDM=gestational diabetes mellitus

**Table 5: Neonatal outcomes.**

| **Outcome/Reference** | **Characteristics of Outcome, unit** | **Intervention group** | **n(total) intervention group** | **Control group(s)** | **n(total) control group** | **Significance** |
| --- | --- | --- | --- | --- | --- | --- |
| **Transfer to higher level of care** | | | | | | |
| Yang et al. 2018 ^30^ | n (%) | 2 (3.51) | n=57 | 1 (2.0), no GDM  4 (8.0), GDM | n=50, no GDM  n=50, GDM | p=0.637 |
| Mackillop et al. 2018 ^29^ | n (%) | 5 (5.0) | n=101 | 12 (12.1) | n=99 | p=0.08 |
| Miremberg et al. 2018 ^22^ | n (%) | 6 (10) | n=60 | 7 (11.6) | n=60 | p>0.99 |
| Borgen et al. 2019 ^26^ | Yes, n (%) | 14 (12.5) | n=112 | 20 (16.5) | n=121 | p=0.38 |
|  | No, n (%) | 98 (87.5) |  | 101 (83.5) |  |  |
| **Birth weight** | | | | | | |
| Yang et al. 2018 ^30^ | g | 3199.47 (389.53) | n=57 | 3200.80 (493.43), no GDM  3169.60 (524.03), GDM | n=50 | p=0.988 |
| Mackillop et al. 2018 ^29^ | g | 3440 (516) | n=101 | 3338 (559) | n=101 | p=0.18 |
| Miremberg et al. 2018 ^22^ | g | 3097.8 (548.2) | n=60 | 3203.3 (414.6) | n=60 | p=0.878 |
| Borgen et al. 2019 ^26^ | ≤4000 g | 100 (89.3) | n=112 | 106 (87.6) | n=121 | p=0.69 |
|  | >4000 g | 12 (10.7) |  | 15 (12.4) |  |  |
| **Fetal Macrosomia** | | | | | | |
| Guo et al. 2019 ^28^ | n (%) | 4 (6.3) | n=64 | 6 (10.0) | n=60 | p=0.295 |
| Yang et al. 2018 ^30^ | n (%) | 2 (3.51) | n=57 | 3 (6), no GDM  2 (4), GDM | n=50 | p=0.542 |
| **LGA** | | | | | | |
| Mackillop et al. 2018 ^29^ | n (%) | NR | n=101 | NR | n=102 | p>0.05 |
| Miremberg et al. 2018 ^22^ | n (%) | 7 (11.6) | n=60 | 7 (11.6) | n=60 | p>0.99 |
| **Hypoglycemia of the Newborn** | | | | | | |
| Guo et al. 2019 ^28^ | n (%) | 1 (1.6) | n=64 | 2 (3.3) | n=60 | p=0.185 |
| Mackillop et al. 2018 ^29^ | n (%) | 31 (32.3) | n=96 | 25 (26.9) | n=93 | p=0.42 |
| Miremberg et al. 2018 ^22^ | n (%) | 2 (3.3) | n=60 | 1 (1.7) | n=60 | p>0.99 |
| Yang et al. 2018 ^30^ | n (%) | 0 (0) | n=57 | 0 (0.0), no GDM  2 (4.0%), GDM | n=50 | NR |

All data shown as number (%) or mean (standard deviation or 95% confidence interval); results considered significant p<0.05.

GDM=gestational diabetes mellitus, LGA=large for gestational age, NR=not reported.
